# Exercise-Based Hybrid Rehabilitation in Diabetic Dyslipidemia: A Systematic Review of Functional and Metabolic Outcomes

**DOI:** 10.7759/cureus.111951

**Published:** 2026-07-02

**Authors:** Janhavee S Brahmadande, Poovishnu T Devi, Rushikesh S Patil, Kiran S Dhaygude

**Affiliations:** 1 Department of Cardiopulmonary Sciences, Krishna College of Physiotherapy, Krishna Vishwa Vidyapeeth (Deemed To Be University), Karad, IND

**Keywords:** aerobic exercise, diabetic dyslipidemia, exercise training, flexibility exercise, functional capacity, hybrid rehabilitation, obesity indices, quality of life, rehabilitation, resistance exercise

## Abstract

Diabetic dyslipidemia is a prevalent metabolic disorder linked to obesity, diminished functional capacity, elevated cardiovascular risk, and poorer health-related quality of life (HRQoL) in people diagnosed with diabetes mellitus. Non-pharmacological approaches, particularly exercise-based rehabilitation and nutritional management, have gained increasing importance in improving metabolic and physical health outcomes in this population. This systematic review examined the effects of integrated exercise training programs and nutritional therapies on obesity measures, functional capacity, lipid profile, and quality of life in patients with diabetic dyslipidemia.

An all-inclusive literature analysis was undertaken incorporating PubMed, Google Scholar, Scopus, ScienceDirect, and Web of Science with the aim to identify articles published between 2002 and 2025. The review focused primarily on randomized controlled trials, clinical trials, observational studies, cross-sectional studies, and meta-analyses of exercise, rehabilitation, and nutritional interventions. The evaluation of bias risk was conducted using the Revised Cochrane Risk of Bias Tool for Randomized Trials (RoB 2) and Appraisal Tool for Cross-Sectional Studies (AXIS), as per the study design. In total, 12 studies fulfilled the requirements for inclusion.

The results showed that integrated exercise interventions, such as aerobic training, resistance exercises, combined exercise programs, and home-based rehabilitation, produced significant improvements in lipid levels, body composition, aerobic fitness, functional performance, obesity-related parameters, and HRQoL. Dietary interventions, including calorie-restricted diets and lifestyle modification strategies, also contributed positively to glycemic regulation, lipid metabolism, and reduction of cardiovascular risk factors. Most research in this review showed moderate-to-high methodological quality and generally a low risk of bias.

This review concludes that combined exercise training and nutritional intervention programs are safe and advantageous for ameliorating obesity indices, functional capacity, lipid profile, and general well-being within people who have diabetic dyslipidemia. Therefore, multidisciplinary rehabilitation techniques may be essential for the long-term control and avoidance of problems associated with diabetic dyslipidemia.

## Introduction and background

Diabetic dyslipidemia is a typical lipid abnormality noticed among patients presenting with diabetes mellitus and is characterized by elevated triglyceride levels, reduced high-density lipoprotein (HDL) cholesterol, and increased small dense low-density lipoprotein (LDL) particles. This abnormal lipid profile is highly atherogenic and significantly promotes the progression of cardiovascular diseases, which remain a significant cause of morbidity and mortality among individuals with diabetes [1]. The etiology of diabetic dyslipidemia is primarily related to insulin resistance and impaired insulin action, which alter normal lipid metabolism. Accelerated lipolysis within adipose tissue prompts a surplus of free fatty acids to enter the bloodstream, stimulating hepatic production of triglyceride-rich very LDLs. Reduced lipoprotein lipase activity further impairs clearance of triglyceride-rich lipoproteins, resulting in hypertriglyceridemia. Additionally, the formation of small dense LDL particles and the reduction of HDL cholesterol levels are stimulated by cholesteryl ester transfer protein-mediated lipid exchange, yielding the hallmark lipid triad of diabetic dyslipidemia [2].

The prevalence of diabetic dyslipidemia has increased globally alongside the rising burden of diabetes mellitus. Epidemiological studies report that nearly 60%-90% of individuals with diabetes exhibit at least one lipid abnormality. Diabetic dyslipidemia is highly prevalent in India and is closely linked to a greater chance of cardiovascular problems such as peripheral vascular disease and coronary artery disease [3]. Multiple components affect the progress and evolution of diabetic dyslipidemia, including advancing age, obesity, sedentary lifestyle, poor dietary habits, family history of diabetes, and poor glycemic control. Central obesity plays a particularly important role by increasing insulin resistance and free fatty acid release, thus worsening lipid abnormalities. Waist circumference and waist-hip ratio have exhibited stronger connections with dyslipidemia than BMI alone [4].

Diabetic dyslipidemia also negatively affects functional capacity and health-related quality of life (HRQoL). Lipid abnormalities contribute to endothelial dysfunction, impaired skeletal muscle metabolism, fatigue, reduced exercise tolerance, and decreased physical performance. In addition, the chronic burden of metabolic disease adversely affects psychological well-being and daily functioning, thereby reducing overall quality of life [5]. Persistent diabetic dyslipidemia corresponds to several comorbidities such as hypertension, cardiovascular disease, chronic kidney disease, and peripheral arterial disease. Poorly controlled lipid abnormalities further increase morbidity and mortality among individuals with diabetes, emphasizing the importance of early identification and management [6]. Management of diabetic dyslipidemia includes lifestyle modifications, dietary regulation, physical activity, weight management, glycemic control, and pharmacological therapy, with statins remaining the first-line treatment because of their proven cardiovascular benefits [7].

Hybrid rehabilitation refers to a multidisciplinary rehabilitation approach that combines two or more therapeutic strategies, most commonly aerobic exercise, resistance training, dietary modification, lifestyle counseling, and patient education, to improve both metabolic and functional outcomes. In patients with diabetic dyslipidemia, hybrid rehabilitation aims not only to optimize lipid metabolism and glycemic control but also to enhance physical fitness, functional capacity, body composition, and HRQoL. Compared with single-modality interventions, hybrid rehabilitation may provide greater overall clinical benefits through its combined physiological effects.

Although pharmacological therapy, particularly statins, remains the cornerstone of dyslipidemia management, medications alone cannot adequately address obesity, reduced physical fitness, muscle weakness, impaired functional capacity, or sedentary lifestyle, which commonly coexist in patients with diabetic dyslipidemia. Furthermore, residual cardiovascular risk often persists despite optimal lipid-lowering therapy. Therefore, comprehensive lifestyle interventions, particularly structured exercise-based rehabilitation, are recommended as an essential adjunct to pharmacological treatment.

Exercise plays a central role in the management of diabetic dyslipidemia by improving insulin sensitivity, increasing skeletal muscle glucose uptake, enhancing lipoprotein lipase activity, reducing triglyceride concentrations, increasing HDL cholesterol, and lowering cardiovascular risk. Aerobic exercise primarily improves cardiovascular fitness and lipid metabolism, whereas resistance exercise enhances muscle mass, strength, and insulin sensitivity. The combination of these exercise modalities may produce greater improvements in metabolic control, functional capacity, obesity indices, and quality of life than either intervention alone.

Hence, this systematic review aims to evaluate the effects of exercise-based hybrid rehabilitation, including combined aerobic exercise, resistance training, dietary modification, and lifestyle interventions, on functional and metabolic outcomes in individuals with diabetic dyslipidemia. The review specifically examines their effects on lipid profile, obesity indices, functional capacity, and HRQoL while identifying current evidence gaps and future research directions.

Given the limited number of studies specifically evaluating exercise-based hybrid rehabilitation in diabetic dyslipidemia, the available evidence was synthesized to provide a comprehensive overview of current knowledge regarding functional and metabolic outcomes.

## Review

Methodology

A systematic review was undertaken pursuant to an evidence-based review methodology, incorporating clinical trials, randomized controlled trials (RCTs), cross-sectional studies, meta-analyses, and observational studies. The Revised Cochrane Risk of Bias Tool for Randomized Trials (RoB 2) was incorporated to analyze RCTs and the Appraisal Tool for Cross-Sectional Studies (AXIS) tool for cross-sectional research to examine the risk of bias.

PICO Framework

The population (P) included adults with diabetic dyslipidemia. The intervention (I) comprised exercise-based rehabilitation, including aerobic exercise, resistance training, and hybrid rehabilitation interventions. The comparator (C) included standard care, no intervention, or alternative rehabilitation approaches where applicable. The outcomes (O) were functional outcomes (e.g., functional capacity and quality of life) and metabolic outcomes (e.g., lipid profile, BMI, waist circumference, and glycemic control).

Search Strategies

An extensive literature exploration was executed to identify studies evaluating the outcomes of exercise training, dietary interventions, and rehabilitation programs on obesity and functional capacity, as well as HRQoL, among individuals with diabetic dyslipidemia. Relevant literature published between January 2002 and December 2025 was identified through electronic databases, including PubMed, Google Scholar, Scopus, ScienceDirect, and Web of Science. The literature search and study selection were completed in April 2026. The search strategy was adapted for each database using appropriate combinations of Medical Subject Headings (MeSH), keywords such as “dyslipidemia”, “diabetic dyslipidemia”, “exercise training", “resistance training", “aerobic exercise", “functional capacity", “obesity indices”, "quality of life”, “6-minute walk test”, “rehabilitation”, and “nutrition intervention", and Boolean operators to maximize the retrieval of relevant studies. In addition, lists of references for selected research studies were manually screened to identify further relevant publications. The review included English-language RCTs, observational studies, cross-sectional studies, and narrative reviews. To assess the risk of bias, RoB 2 was used for RCTs, whereas the AXIS tool was used for cross-sectional studies.

Study Selection

Following the removal of duplicate records, the retrieved studies underwent title and abstract screening based on the predefined eligibility criteria. Full-text articles of potentially relevant studies were subsequently assessed for inclusion in the review. The study selection process was conducted in accordance with the Preferred Reporting Items for Systematic Reviews and Meta-Analyses (PRISMA) 2020 guidelines, and any uncertainties regarding study eligibility were resolved through discussion and consensus.

Data Extraction

Data were extracted using a standardized data extraction approach. Information collected from each study included the author, publication year, study design, sample size, participant characteristics, intervention details, outcome measures, key findings, and conclusions relevant to the objectives of this review.

Assessment of Risk of Bias

All selected studies underwent a risk-of-bias assessment to ensure the credibility and reliability of the synthesized evidence. A detailed evaluation of the included literature was conducted using established research appraisal tools to confirm the validity of the reported findings.

Depending on the RoB 2 assessment, most of the encompassed RCTs demonstrated a low risk of bias regarding the randomization process, missing outcome data, and selective reporting of results. However, concerns were identified in all studies related to deviations from intended interventions and outcome assessment, primarily because blinding participants and assessors is difficult in exercise- and rehabilitation-based interventions. Consequently, the complete risk of bias for the RCTs was defined as “some concerns," indicating acceptable methodological quality with minor issues related to performance and detection bias.

Inclusion Criteria

Studies involving adults with type 2 diabetes mellitus (T2DM) and diabetic dyslipidemia were included. Studies evaluating exercise-based rehabilitation interventions and relevant metabolic or functional outcomes were considered eligible. The analysis was confined to research published up until 2025. Only studies published up to December 2025 were considered eligible, and only articles found on digital portals such as Google Scholar, ResearchGate, PEDro, MEDLINE, and PubMed were taken into consideration.

Exclusion Criteria

Studies involving non-diabetic dyslipidemia, non-English publications, conference abstracts, editorials, case reports, animal studies, and studies lacking relevant outcome measures were excluded. Furthermore, the review excluded studies that did not align with the predetermined inclusion criteria for a thorough assessment of therapeutic interventions and diagnostic systems.

Quality Assessment

Standardized risk-of-bias assessment tools were used to determine the scientific rigor of the included research as per their respective study designs. For RCTs, the RoB 2 was deployed to evaluate potential bias originating from the randomization process, deviations from assigned interventions, missing outcome data, outcome measurement, and the selection of reported results. The AXIS was applied to cross-sectional studies and evaluated domains, including study objectives, sample selection, measurement validity, statistical analysis, reporting quality, ethical considerations, and potential non-response bias.

Overall, most RCTs displayed low- to moderate-bias concerns, with common limitations related to participant and assessor blinding, concurrently with occasional issues with incomplete outcome data. Similarly, cross-sectional studies generally demonstrated a low risk of bias, albeit with some concerns with respect to sample representativeness and non-response bias. Despite these limitations, the majority of the included studies exhibited moderate to good methodological quality, supporting their suitability for inclusion in this review.

**Figure 1:**
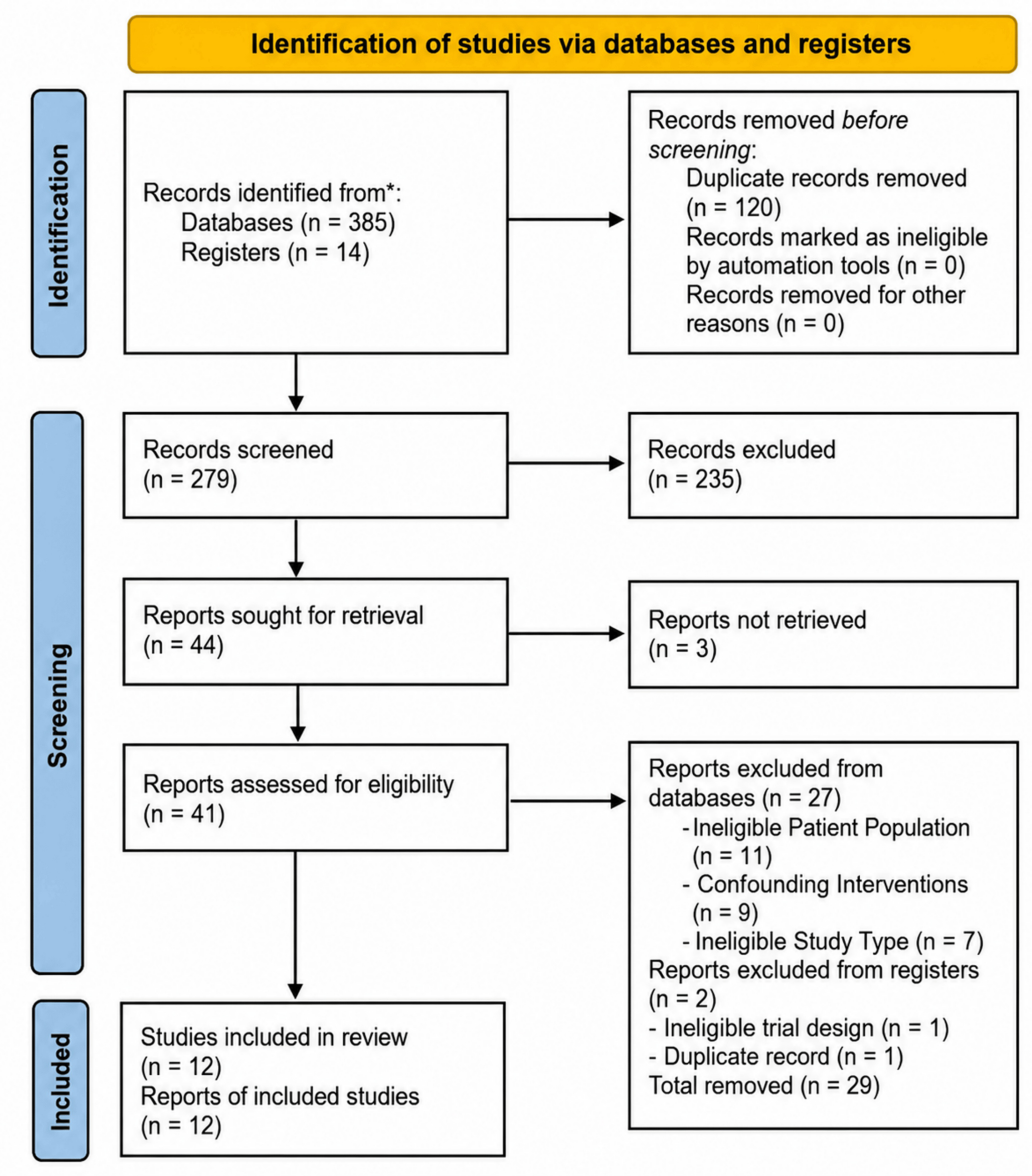
PRISMA 2020 flow diagram of study selection process *Refers to the identification of records from databases and registers PRISMA, Preferred Reporting Items for Systematic Reviews and Meta-Analyses.

A total of 399 records were identified through database searching (n=385) and registers (n=14). After removing 120 duplicate records, 279 records remained for screening. Of these, 235 records were excluded based on title and abstract screening. Forty-four reports were sought for retrieval, of which three reports could not be retrieved. The remaining 41 full-text articles were assessed for eligibility. After excluding 29 reports due to ineligible patient populations, confounding interventions, inappropriate study designs, duplicate records, and studies from registers, 12 studies met the inclusion criteria. They were included in the systematic review, as given in Figure 1.

This systematic review was conducted in accordance with the PRISMA 2020 guidelines. However, the review protocol was not prospectively registered, which is acknowledged as a methodological limitation.

Data Synthesis

Owing to substantial heterogeneity in study designs, participant characteristics, intervention protocols, outcome measures, and follow-up durations, a quantitative meta-analysis was not considered appropriate. Therefore, the findings were synthesized using a qualitative narrative approach.

Results

In total, 12 articles were included that were constructed on the predefined inclusion criteria, providing a comprehensive summary of the available evidence regarding exercise-based rehabilitation interventions and their functional and metabolic outcomes in diabetic dyslipidemia.

The studies summarized in the table demonstrate the significant burden of dyslipidemia among individuals with T2DM and other at-risk populations. Cross-sectional studies consistently reported a high prevalence of dyslipidemia and its association with obesity, poor glycemic control, reduced physical fitness, and adverse musculoskeletal changes. RCTs showed that various exercise interventions, including aerobic training, resistance training, hybrid exercise programs, aquatic exercise, and lifestyle modification programs, produced significant improvements in lipid profile parameters such as LDL, HDL, total cholesterol, and triglycerides. Additionally, studies highlighted that greater exercise volume and sustained participation resulted in more favorable metabolic outcomes, whereas discontinuation of exercise reversed these benefits. Overall, the evidence supports regular exercise as an effective non-pharmacological strategy for improving lipid profiles, reducing cardiovascular risk, and enhancing metabolic health in individuals with dyslipidemia and type 2 diabetes, as given in Table 1.

**Table 1:** Overview of studies included in the review GI, glycemic index; HDL-C, high-density lipoprotein cholesterol; LDL, low-density lipoprotein; RCT, randomized controlled trial; T2DM, type 2 diabetes mellitus; TC, total cholesterol; TG, triglyceride.

Author(s)	Year	Study design	Sample size (n)	Intervention	Outcome measures	Results	Conclusion
Yigit et al. [5]	2025	Cross-sectional study	144	Observational comparison (T2DM with vs without dyslipidemia)	Skeletal muscle alterations, dyslipidemia status	Dyslipidemia associated with worse muscle alterations in T2DM	Dyslipidemia worsens musculoskeletal health in T2DM.
Suder et al. [8]	2024	RCT	140	Aerobic + resistance exercise + high-protein low-GI diet	Irisin, omentin, lipid profile	Significant improvement in lipid and metabolic markers	Combined exercise + diet improves dyslipidemia and metabolic health.
Alghadir et al. [9]	2024	RCT	50	Supervised aerobic training	TC, LDL, HDL, TG	Significant improvement in lipid profile	Aerobic training improves dyslipidemia in patients with diabetes.
Nandasena et al. [10]	2023	Cross-sectional study	366	Dyslipidemia prevalence	Population-based survey	High prevalence observed	Dyslipidemia is a major public health burden.
Al Quran et al. [11]	2022	Cross-sectional study	870	Assessment of dyslipidemia in patients with T2DM	Lipid profile (TC, LDL, HDL, TG), associated risk factors	High prevalence of dyslipidemia in T2DM; linked with poor glycemic control and obesity	Dyslipidemia is highly prevalent in T2DM and associated with metabolic risk factors.
Zhou et al. [12]	2021	Cross-sectional study	776	Physical fitness assessment (no intervention)	Dyslipidemia risk, fitness level	Lower fitness associated with higher dyslipidemia risk	Physical fitness is inversely related to dyslipidemia.
Costa et al. [13]	2019	RCT	69	Water-based aerobic and resistance training	Total cholesterol, HDL-C, LDL, triglycerides	Both aerobic and resistance aquatic training improved lipid profiles, with reductions in triglycerides and LDL with increased HDL	Water-based exercise is an effective non-pharmacological treatment for dyslipidemia in women.
Wankhade et al. [14]	2018	Cross-sectional study	300	No intervention (industrial workers risk assessment)	Lipid profile, BMI, diabetes, hypertension, tobacco use	Dyslipidemia prevalence ~50.7%; strong association with risk factors	Dyslipidemia is highly prevalent and linked to modifiable risk factors.
Shakil-Ur-Rehman et al. [15]	2017	RCT	40	Supervised aerobic exercise	HDL, LDL, total cholesterol	↑HDL and ↓LDL	Aerobic exercise improves lipid profile in T2DM.
Shrivastava et al. [16]	2017	RCT	98	Worksite lifestyle intervention, including exercise	Body weight, fat, cardiometabolic risk	Reduced cardiometabolic risk and body fat	Workplace exercise improves metabolic health.
Slentz et al. [17]	2007	RCT	240	Plasma lipoproteins	Exercise training vs detraining	Exercise improved HDL/TG; detraining reversed the effects	Exercise improved HDL/TG; detraining reversed effects
Kraus et al. [18]	2002	RCT	111	Different amounts and intensities of exercise training	Plasma lipoproteins, HDL, LDL, triglycerides	Higher amounts of exercise produced greater improvements in lipid profile	Regular exercise, especially higher energy expenditure, improves dyslipidemia and cardiovascular risk factors.

Assessment of Risk of Bias

All selected studies underwent a risk-of-bias assessment to ensure the credibility and reliability of the synthesized evidence. A detailed evaluation of the included literature was conducted using established research appraisal tools to confirm the validity of the reported findings.

Depending on the RoB 2 assessment, most of the encompassed RCTs demonstrated a low risk of bias regarding the randomization process, missing outcome data, and selective reporting of results. However, concerns were identified in all studies related to deviations from intended interventions and outcome assessment, primarily because blinding participants and assessors is difficult in exercise- and rehabilitation-based interventions. Consequently, the complete risk of bias for the RCTs was defined as “some concerns," indicating acceptable methodological quality with minor issues related to performance and detection bias, as given in Table 2.

**Table 2:** Risk-of-bias assessment using the RoB 2 RoB 2, Revised Cochrane Risk of Bias Tool for Randomized Trials.

Author(s)	Year	Bias arising from the randomization process	Bias due to deviations from intended interventions	Bias due to missing outcome data	Bias in the measurement of the outcome	Bias in the selection of the reported result	Overall risk of bias
Suder et al. [8]	2024	Low risk	Low risk	Some concerns	Low risk	Low risk	Low risk
Alghadir et al. [9]	2024	Low risk	Low risk	Low risk	Low risk	Low risk	Low risk
Costa et al. [13]	2019	Low risk	Low risk	Some concerns	Low risk	Low risk	Low risk
Shakil-Ur-Rehman et al. [15]	2017	Some concerns	Low risk	Some concerns	Low risk	Low risk	Some concerns
Shrivastava et al. [16]	2017	Some concerns	Some concerns	Some concerns	Low risk	Some concerns	Some concerns
Slentz et al. [17]	2007	Low risk	Low risk	Low risk	Low risk	Low risk	Low risk
Kraus et al. [18]	2002	Low risk	Low risk	Low risk	Low risk	Low risk	Low risk

The AXIS tool evaluation indicated that the included cross-sectional studies generally possessed good methodological quality. All studies explicitly specified their aims and objectives, applied appropriate study designs, adequately defined the target population, applied suitable sampling methods, utilized validated outcome measurement tools, and conducted appropriate statistical analyses. In addition, the studies provided sufficient details regarding methodology, results, ethical considerations, discussions, and conflict-of-interest disclosures. Yet, certain restrictions were detected concerning the representativeness of the selected samples and the possibility of non-response bias. Moreover, certain research failed to provide thorough details about non-respondents or clearly describe measures taken to address non-response issues. Overall, the observational studies included in the review were considered to have a low risk of bias, with selection bias and non-response bias being the primary concerns, as given in Table 3.

**Table 3:** Quality assessment of included cross-sectional studies using the AXIS AXIS, Appraisal Tool for Cross-Sectional Studies.

AXIS appraisal question	Yigit et al. [5]	Nandasena et al. [10]	Al Quran et al. [11]	Zhou et al. [12]	Wankhade et al. [14]
Were the aims/objectives of the study clear?	Yes	Yes	Yes	Yes	Yes
Was the study design appropriate for the aims?	Yes	Yes	Yes	Yes	Yes
Was the sample size justified?	Yes	Yes	Yes	Yes	No
Was the target population clearly defined?	Yes	Yes	Yes	Yes	Yes
Was the sampling frame appropriate?	Yes	Yes	Yes	Yes	Yes
Was the selection process representative?	Yes	Yes	Yes	Yes	Some concerns
Were measures taken to address non-responders?	No	No	No	No	No
Were the outcome variables measured appropriately?	Yes	Yes	Yes	Yes	Yes
Were the tools piloted/published previously?	Yes	Yes	Yes	Yes	Yes
Was statistical significance clearly determined?	Yes	Yes	Yes	Yes	Yes
Were the methods described in enough detail?	Yes	Yes	Yes	Yes	Yes
Were the basic data adequately described?	Yes	Yes	Yes	Yes	Yes
Is there concern regarding non-response bias?	Some concern	Some concern	Some concern	Some concern	Some concern
Was the info about non-respondents described?	No	No	No	No	No
Were the results internally consistent?	Yes	Yes	Yes	Yes	Yes
Were all method-defined results presented?	Yes	Yes	Yes	Yes	Yes
Were discussions and conclusions justified?	Yes	Yes	Yes	Yes	Yes
Were the study limitations discussed?	Yes	Yes	Yes	Yes	Yes
Were funding/conflicts of interest reported?	Yes	Yes	Yes	Yes	Yes
Was ethical approval/informed consent attained?	Yes	Yes	Yes	Yes	Yes

Discussion

Although the primary focus of this review is exercise-based rehabilitation, relevant observational and epidemiological studies were also considered to provide clinical context for interpreting the available evidence. The present review collected data through 12 investigations, including RCTs and cross-sectional studies, examining dyslipidemia due to T2DM, its associated risk factors, and the effects of lifestyle-based interventions such as exercise and dietary modification. Overall, the findings demonstrate a consistent pattern: dyslipidemia is highly prevalent in T2DM and related populations, is strongly influenced by modifiable metabolic and lifestyle factors, and can be significantly improved through structured exercise and combined lifestyle interventions.

Across the cross-sectional studies, dyslipidemia was consistently identified as a major metabolic abnormality in both diabetic and general populations. Al Quran et al. reported a high prevalence of dyslipidemia in patients with T2DM, strongly associated with impaired glycemic control, obesity, and adverse metabolic risk profiles [11]. Similarly, Wankhade et al. highlighted a substantial burden of dyslipidemia in industrial workers, reinforcing the role of occupational lifestyle factors and behavioral risks such as tobacco use, physical inactivity, and increased BMI [14]. These results collectively indicate that dyslipidemia is not restricted to clinical diabetic populations but is also widespread in at-risk community and occupational groups. Auxiliary to this, Nandasena et al. demonstrated that dyslipidemia represents a significant public health burden at the population level, emphasizing the need for early detection and preventive strategies [10]. Zhou et al. added an important dimension by showing that lower physical fitness is significantly associated with higher dyslipidemia risk, reinforcing physical inactivity as a key modifiable determinant of lipid abnormalities [12]. The collective findings of these investigations show that dyslipidemia is strongly influenced by lifestyle behaviors and population-level risk factors.

In addition, Yigit et al. expanded the understanding of dyslipidemia beyond cardiovascular risk by showing its relation with skeletal muscle alterations in individuals with T2DM. Consequently, dyslipidemia may contribute to musculoskeletal dysfunction and functional decline, highlighting its systemic impact. Although these cross-sectional findings cannot establish causality, they emphasize the broader clinical implications of lipid abnormalities in metabolic disease. Evidence from RCTs strengthens the role of structured exercise interventions in improving dyslipidemia [5]. Kraus et al. demonstrated a dose-response correlation between physical activity volume and lipid improvement, with higher levels of physical activity producing greater reductions in triglycerides and LDL cholesterol while improving HDL cholesterol [18]. Similarly, Slentz et al. confirmed that designed exercise training enhances lipid profiles, whereas detraining reverses these benefits, highlighting the necessity of sustained physical activity for maintaining metabolic health [17].

Costa et al. showed that water-based aerobic and resistance training significantly improved lipid profiles in women with dyslipidemia, supporting the effectiveness of alternative exercise modalities [13]. Alghadir et al. and Shakil-Ur-Rehman et al. both demonstrated that supervised aerobic exercise leads to significant improvements in LDL, HDL, and triglyceride levels in diabetic populations [9,15]. Shrivastava et al. extended these findings by showing that workplace-based lifestyle interventions reduce body fat and overall cardiometabolic risk, highlighting the effectiveness of structured interventions in real-world settings [16].

Furthermore, Suder et al. demonstrated that combining aerobic and resistance exercise with a dietary modification (high-protein, low-glycemic index diet) produces significant improvements in lipid and metabolic biomarkers, suggesting that multimodal interventions may be more beneficial than exercise alone [8]. Collectively, the RCTs consistently support exercise and lifestyle modification as effective non-pharmacological strategies for managing dyslipidemia. When integrating evidence across all 12 studies, a clear and coherent pattern emerges. Cross-sectional studies (Yigit et al., Nandasena et al., Al Quran et al., Zhou et al., and Wankhade et al.) establish that dyslipidemia is highly prevalent and strongly associated with modifiable lifestyle, metabolic, and functional factors [5,10-12,14]. In contrast, RCTs (Suder et al., Alghadir et al., Costa et al., Shakil-Ur-Rehman et al., Shrivastava et al., Slentz et al., and Kraus et al.) highlight that these potential risk variables can be effectively targeted through structured exercise and combined lifestyle interventions, leading to significant improvements in lipid profiles [8,9,13,15-18]. This integration of observational and interventional evidence strengthens the overall conclusion that dyslipidemia in T2DM is both a widespread metabolic disorder and a modifiable condition. It highlights the importance of lifestyle interventions, particularly physical activity, as a cornerstone of dyslipidemia management alongside pharmacological treatment when required. 

Although this review uses the term "hybrid rehabilitation," not all included studies evaluated comprehensive hybrid rehabilitation programs. Several studies assessed aerobic exercise alone, resistance training alone, or observational outcomes without multidisciplinary interventions. Therefore, the findings should be interpreted in the context of heterogeneous rehabilitation approaches.

This systematic review has several limitations. The available literature specifically evaluating exercise-based hybrid rehabilitation in diabetic dyslipidemia remains limited; therefore, studies with different methodological designs were included to provide a comprehensive overview of the current evidence. Additionally, the included studies were heterogeneous in design, interventions, outcome measures, and follow-up duration, and several had relatively small sample sizes, limiting direct comparisons and long-term conclusions. Despite these limitations, the available evidence suggests that structured exercise and lifestyle interventions can improve metabolic and functional outcomes in individuals with diabetic dyslipidemia. The heterogeneity of the included studies precluded quantitative meta-analysis and prevented reporting of pooled effect estimates, including overall p-values and 95% confidence intervals.

Future research should focus on large-scale, long-term RCTs using standardized intervention protocols and outcome measures to strengthen the evidence base. The heterogeneity of the included studies also limited the feasibility of performing a quantitative meta-analysis.

This review explains that dyslipidemia is a common and clinically significant metabolic disturbance closely associated with lifestyle and physical fitness levels. While cross-sectional studies establish its burden and associated risk factors, RCTs confirm that structured aerobic, resistance, and combined lifestyle interventions can significantly improve lipid profiles. This systematic review comprehensively synthesizes the current evidence on exercise-based hybrid rehabilitation in diabetic dyslipidemia, providing clinically relevant insights into its effects on functional and metabolic outcomes. However, the findings should be interpreted with caution due to heterogeneity in study designs, exercise protocols, outcome measures, and sample sizes, which limited direct comparisons and generalizability. Furthermore, the inclusion of only English-language published studies may have introduced publication and language bias.

## Conclusions

Exercise-based rehabilitation programs, particularly those combining aerobic exercise, resistance training, and dietary modification, are effective in improving obesity indices, functional capacity, lipid profile, and HRQoL in individuals with diabetic dyslipidemia. Exercise interventions combining aerobic and resistance training, particularly when integrated with dietary modification, help reduce body weight, elevate lipid profiles, enhance physical performance, and promote overall well-being. Overall, the findings suggest that structured multidisciplinary lifestyle rehabilitation should be considered an important component in the management of diabetic dyslipidemia.
